# The naïve language expert: introduction to the research topic

**DOI:** 10.3389/fpsyg.2013.00526

**Published:** 2013-08-20

**Authors:** Jutta L. Mueller, Claudia Männel

**Affiliations:** ^1^Psycho/Neurolinguistics Group, Institute of Cognitive Science, University of OsnabrückOsnabrück, Germany; ^2^Department of Neuropsychology, Max Planck Institute for Human Cognitive and Brain SciencesLeipzig, Germany

Since the first seminal reports of young infants' abilities to use both acoustic (e.g., Mandel et al., 1994) and statistical cues (e.g., Saffran et al., 1996) to structure, categorize, and memorize linguistic units from their speech input, the quest for capturing infants' abilities and limitations in the discovery of basic elements and regularities in speech has attracted a lot of attention. While many important findings have been unveiled using sophisticated behavioral methods that allow to measure infants' discrimination of familiar vs. unfamiliar speech sounds, the field has gained a new momentum with the advent of techniques, such as event-related brain potentials (ERPs) or functional near-infrared spectroscopy (fNIRS), which allow to measure discrimination even in the absence of overt behavioral responses. After the first excitement about infants' amazing abilities, new challenges have emerged, for example, the question how different input cues interact, how learner variables, such as bilingual language input, contribute to learning mechanisms, or how low-level learning mechanisms contribute to higher-order language learning, such as word learning or sentence comprehension.

The goal of the current Research Topic is to provide a cutting-edge snapshot of this active research field integrating original research papers using both behavioral and neurophysiological techniques with review articles providing ideas for general frameworks capturing those findings.

Three reviews and one methods article offer global and thought-provoking views on basic principles and computational mechanisms that are operative in early language learning. Seidl and Cristia (2012) provide an overview of research on the discovery of allophony vs. phonemic differences in early infancy and discuss mechanisms supporting this distinction. Bortfeld et al. (2013) make a case for using neurophysiological methods, such as ERPs and fNIRS to investigate two factors they consider basic ingredients for early language learning, namely salience and familiarity. Krogh et al. (2012) provide a timely review of statistical learning across modalities and outline different types of constraints and underlying learning mechanisms. Arciuli and Torkildsen (2012) provide evidence for a close interaction between statistical learning and language processing in normal and impaired language acquisition and call for longitudinal research programs shedding light on this relationship.

Two of the original research papers applied neurophysiological methods. Minagawa-Kawai et al. (2013) report an fNIRS study on the emergence of phonotactic abilities in a cross-linguistic sample of infants. The authors report a null-result and discuss potential methodological pitfalls when using fNIRS. Kooijman et al. (2013) used ERPs measured at the age of 7 months as a predictor of later language skills showing the potential sensitivity and meaningfulness of neurophysiological measures with respect to inter-individual differences across language development.

Another set of research articles focuses on the contribution of prosodic information to the perception of sentential structure. Wellmann et al. (2012) evaluate the role of different prosodic boundary cues in German-learning infants' discrimination of coordinate noun phrases, showing that two out of three cues are sufficient for 8-month-olds to solve this task. For the same age, Bernard and Gervain (2012) show that French-learning infants use prosodic prominence and word frequency as signals to word order in an artificial language.

The largest group of papers deals with specific questions related to speech segmentation. Bosch et al. (2013) investigate word segmentation in 6- and 8-month-olds in previously underinvestigated, syllable-timed languages (i.e., Spanish and Catalan) and provide evidence for the early emergence of this ability in monolinguals and bilinguals. For English-learning infants, Thiessen and Erickson (2012) show that this ability emerges even at 5 months if artificial-language units are marked by transitional probabilities and word stress, and that infants' segmentation is guided by transitional probabilities if both information types are placed in conflict. Yurovsky et al. (2012) also study regularities signaling word-like units in child-directed speech, that is, position and onset cues in naming frames. The authors report that in an artificial language either regularity is sufficient to trigger speech segmentation and subsequent word learning in adults. Mintz (2013) is interested in the question when infants are able to segment morphosyntactic endings from verb stems and provides evidence that this happens starting from the first half of the second year of life. Graf Estes (2012) demonstrates that infants at 11 and 17 months recognize words across acoustic variations after successful statistical segmentation and at the older age even apply these generalizations as labels of new objects.

Finally, our Research Topic contains one study which analyzes infant speech production during the second year of life. Yamashita et al. (2013) study English- and Japanese-learning children's phonetic inventory across 15, 20, and 24 months and assume adult-like vocal tract structures to be present by 24 months of age for both languages.

As a compendium of current research efforts in the field of early language learning mechanisms we are confident that this Research Topic offers novel and stimulating ideas for those who are new to the field and would like to get a timely overview as well as for experts who are interested in current developments.
